# Participation and engagement of a rural community in Ciclovía: progressing from research intervention to community adoption

**DOI:** 10.1186/s12889-021-11980-6

**Published:** 2021-10-30

**Authors:** Linda K. Ko, Eligio Jimenez, Oralia Cisneros, Emily V. R. Brown, Genoveva Ibarra, Sonia Bishop, Monica Escareño, Luis Serrano-Rubio, Eileen Rillamas-Sun, Jason A. Mendoza, Sarah Sutton

**Affiliations:** 1 Department of Health Systems and Population Health, Hans Rosling Center for Population Health, University of Washington, 3980 15th Avenue NE, UW Mailbox 351621, Seattle, WA 98195 USA; 2 Division of Public Health Sciences, Fred Hutchinson Cancer Research Center, 1100 Fairview Ave. N, M3-B232, Seattle, WA 98109 USA; 3 Community Safety Network, 306 Bolin Dr, Toppenish, WA 98948 USA; 4 Granger School District, 701 E Ave, Granger, WA 98932 USA; 5 Center for Community Health Promotion, Fred Hutchinson Cancer Research Center, 320 N. 16th Street, Sunnyside, WA 98944 USA; 6 Department of Pediatrics, University of Washington School of Medicine, Seattle, USA

**Keywords:** Ciclovía, Rural communities, Physical activity, Safe spaces, Bidirectional learning

## Abstract

**Background:**

Open streets events, where roads are temporarily closed to motorized vehicles, can provide safe spaces for physical activity (PA) and become sustainable community infrastructure. Since 2016, we have collaborated with a rural community to implement an open streets event, named ciclovía. In 2019, ciclovía was adopted as a community-wide program. This paper describes the process of building and progressing a ciclovía from a research intervention to a community-adopted program and participation of a rural community in ciclovía.

**Methods:**

We used community-based participatory research to foster bidirectional learning on how to optimize the content and implementation of ciclovía to be feasible and acceptable for rural communities. The community-academic partnership focused on: 1) understanding the science of ciclovía; 2) learning the implementation process; 3) creating tools to facilitate planning, implementation, and evaluation of ciclovía; and 4) developing transition steps from a research intervention to a community-adopted program.

**Results:**

The progression of the research intervention to community adoption spanned 2 years. First, the partnership met quarterly to discuss the science of ciclovía, its utility, and its adaptation for rural communities. Second, the partnership studied processes that facilitated ciclovía implementation. Third, the partnership created the ciclovía planning guide and tools for communities to establish their own ciclovía. The guide included forming a planning committee, setting meeting and communication plans, marketing and promotion, and selecting evaluation tools. Fourth, the transition steps from research intervention to community adoption included creating roles and responsibilities, implementing ciclovía using the planning guide, and convening listening sessions for improvement on implementation. Community attendance at ciclovía doubled from 189 individuals (126 children and 63 adults) when it was a research intervention to 394 individuals (277 children and 117 adults) when it was a community program.

**Conclusions:**

The progression from a research intervention to a community-adopted program encompasses multiple steps that involve bidirectional learning and partnership with the community. Lessons learned from this study are integrated into a disseminatable ciclovía planning guide.

**Supplementary Information:**

The online version contains supplementary material available at 10.1186/s12889-021-11980-6.

## Background

Open streets initiatives temporarily close streets to motorized traffic so they can be used by individuals for physical activity, such as biking, walking, jogging, and dancing [1–3]. Open streets initiatives are modeled after those held in Bogotá, Columbia, where the term “ciclovía” (or cyclo-via) was coined to describe this type of event [4, 5]. The overall goal of a ciclovía is to enable individuals to reclaim their streets as places that serve as connections between individuals and safe spaces to enjoy active transportation and PA [4, 6]. Ciclovías have become increasingly common in United States (US) cities and towns seeking innovative ways to inspire PA and social cohesion among the community. From 2008 to 2016, approximately 122 US cities hosted at least one open streets event [7]. However, many of these events were in urban and suburban areas where residents were aware of the benefits of a ciclovía and knew how to mobilize their community’s resources and infrastructure to plan the event [7–9].

Children living in rural areas in the US are less likely to be physically active compared to their urban counterparts, in part due to the lack of infrastructure and opportunities for PA [10–14]. Rural communities have fewer parks and general facilities for physical activity, limiting opportunities for children to play and enjoy public spaces [13, 14]. In addition, rural schools tend to lack resources (e.g., equipment, afterschool programs) that facilitate vigorous PA during school time, as well as supervised free play after school [12]. The home environment also influences children’s PA where greater access to media devices (e.g., televisions) and frequent screen time and lower access to sports equipment (e.g., basketball hoops, soccer balls) are associated with physical inactivity [15] and are more prominent among rural families with lower socioeconomic status. These lack of opportunities to be physically active contribute to the obesity rates among rural children where it is higher than their urban counterparts [16].

Ciclovía can provide safe spaces for PA and has potential to become sustainable rural community infrastructure. In April of 2012, we were approached by community members in rural eastern Washington State and were told about the increasing problem of childhood obesity, especially among Hispanics. Together with the community, we applied and received funding in 2013 from the National Institutes of Health to build community infrastructure and capacity to address childhood obesity; this funding led to the formation of a community-academic partnership and established a community advisory board (CAB). The partnership conducted a community-wide needs assessment to understand issues related to the rising cases of childhood obesity [14–17]. A subsequent funding in 2016 led to the development and implementation of a community-wide intervention study entitled “Together We STRIDE (Strategizing Together Rural Interventions for Diet and Exercise).” The purpose of the Together We STRIDE project was to test the effectiveness of a multi-level intervention on nutrition, physical activity, and Body Mass Index (BMI) z-score for children in rural communities [18]. The multi-level intervention consisted of comic books (focused on promoting healthy eating and physical activity), group-based nutrition education and guided PA classes, PA breaks led by teachers during class time, and the ciclovía [18]. This study used a community-based participatory research approach and had a highly engaged CAB that was involved from the beginning of the study to dissemination of results. In 2019, as the study ended, ciclovía was adopted by the community as an annual community-wide program supported by the community. This paper describes the process of developing and progressing ciclovía from a research intervention to a community-adopted program for rural areas.

## Methods

The ciclovía was held in Toppenish, Washington, which is located in a rural agricultural region in eastern Washington State. Based on the Rural-Urban Commuting Area Code (1–10), a standardized approach used to classify rural areas based on town size and commuting patterns to urban areas, Toppenish is classified as 4.2 (large rural community with a population of 10,000 to 49,999) [19]. The Toppenish population is just over 10,000, where 74% of residents are Hispanic and 24% live below the federal poverty level [20]. This study was approved by the Fred Hutchinson Cancer Research Center Institutional Review Boards, and informed consent was obtained from research participants.

### From research to community adoption

We used a community-based participatory research approach to foster bidirectional learning with the community to create the content of the ciclovía, adapt the content to be feasible and acceptable for rural communities, and implement ciclovía, with a focus on sustainability through community adoption. In 2017 and 2018, the ciclovía was a research intervention, while in 2019, the ciclovía became a community-adopted program, supported and administered by the community, with the research team supporting the evaluation. The progression from research intervention to community adoption included four steps: 1) co-learning about the science of ciclovía, 2) learning the implementation process and navigating subsequent iterations, 3) creating tools to facilitate planning, implementation, and evaluation of ciclovía, and 4) developing transition steps from a research intervention to a community-adopted program.

#### Co-learning about the science of ciclovía

The CAB met quarterly to learn and review information about ciclovía as this was initially a new concept for our partnering community. The CAB included 35 community members. The members represented 22 local organizations including schools, community health centers, local health departments, community-based organizations, and community advocates. The CAB received $25 per meeting to compensate for their time and effort. Conversations in these sessions were about ciclovía’s origin from Bogota, Colombia, as a community-led social movement to claim the streets for physical activity; its passage to the US and subsequent adoption by communities with high socioeconomic status; and utility and adaptation of ciclovía to local context with the goal of sustainability. Because Together We STRIDE promoted healthy eating as well as physical activity, the community felt it was important to provide healthy food and beverage options at the event. The community provided healthy food through fruit donations from local farm owners, which included apples, bananas, and oranges. Water was provided as the healthy beverage, using reusable water bottles branded with the study logo for participants to use and take home. These two components helped create synergy with the project’s promotion of PA and healthy eating as interdependent attributes of a healthy, active lifestyle.

#### Learning the implementation process

A smaller steering committee of 10 CAB members was formed within the CAB with those who self-selected to be in the committee based on their availability and interest for more engagement. The steering committee led the planning and the implementation of the ciclovía. The 2017 planning meetings were focused on delineating roles and responsibilities for the community and researchers. The steering committee identified a venue for ciclovía, secured city permits to close streets, enlisted volunteers, and provided input throughout the planning and implementation process. The research team led the planning meetings, incorporated input from the steering committee, coordinated all the activities that would occur during ciclovía, tracked and updated the volunteer list, created the marketing and implementation tools, and led the evaluation of ciclovía.

The steering committee made several adaptations to ciclovía to fit the rural context in three specific ways. First, given that rural communities are more spread out than dense urban areas, the steering committee strategically selected an area with a park and adjacent streets proximal to the town center so that the event included multiple components conducive to PA (e.g., greenspace, crosswalks, and sidewalks connecting to local shops and businesses). Integrating these multiple components helped reinforce the idea that PA is accessible and feasible in the community. It also enabled the community to take advantage of the greenspace for event activities. Second, activity hubs for families (e.g., Zumba, yoga, aerobics) and children (e.g., walk the plank, hula hoops, jump rope) were placed in designated sections of the park and streets. This enabled us to show community members that this space was a community asset that could be shared for all kinds of PA and that the streets could be traveled via active transportation to get to places where people gather for physical and social activities. Third, bicycles and scooters donated by the community were raffled to families throughout the event to increase accessibility of bicycles to children and instill a connection between bicycles and wheels with the theme of ciclovía. To promote safe riding practices, the children who won the raffle were fitted with helmets, since wearing a helmet was not normalized in the community. The planning and implementation process were iterative, where lessons learned from previous years were discussed by the steering committee and consensus was reached to incorporate new elements to improve future ciclovías.

#### Creating tools to facilitate planning, implementation, and evaluation of ciclovía

The steering committee and research team co-created multiple tools to plan and evaluate the ciclovías. Planning tools included standard operating procedures for planning and day-of-event implementation, planning timelines, planning checklists, event maps with locations of the activity stations, and a list of activity stations. Volunteers were assigned to each station. All volunteers were trained on their roles and given information on who to contact if questions arose during the ciclovía. All adolescent volunteers were supervised by adults.

For the 2017 ciclovía, we developed a logistics document to help organize event activities and track roles and responsibilities. The document included event logistics, such as event hours, lists of activity stations, and staff contact information. The document was updated after each ciclovía planning meeting to ensure that what was agreed upon in meetings was documented. We handed out individual copies to staff and volunteers at the ciclovía.

In planning the 2018 ciclovía, we adapted the logistics document into a Planning Checklist to improve usability (see Additional file 1 for Planning Checklist). We organized the checklist by activity and related components: equipment needed, main contact, status, and person(s) responsible for finalizing the activity. Like the logistic document, the checklist was updated after each ciclovía planning meeting to ensure the necessary components of each activity came together.

The evaluation tools included a participant’s survey, a participant count form, and data collection protocol. Data collectors were trained on their assigned tool before the event and received a refresher training on the day of the event to ensure accuracy and consistency.

#### Developing transition steps from a research intervention to a community-adopted program

In 2019, the community advisory board with CAB established transition steps from research intervention to community adoption through four major steps. First, to ensure that it was a community-led event, previous roles and responsibilities were revised and delineated for the community to lead the planning and implementation of the event. The research team provided assistance by scheduling planning meetings, co-managing the planning checklist, updating event flyers and banners, and overseeing the evaluation. Second, the community secured funding and resources through local connections to support the event. Funds were used to complement the donations from the community so that more bicycles, skateboards, and scooters were available through the raffle. A large helmet donation (of 150 helmets) was secured by the community to ensure that all children on wheels were fitted with helmets. Third, a planning guide was created by combining the resources created from previous ciclovías into a step-by-step guide. The manual describes the planning, implementation, and evaluation steps as well as supporting tools and resources to accomplish the plan (Ciclovía: Planning a Rural Open Streets Event; available at the weblink: Ciclovia Planning Manual.) The community used this manual and drew from their own experiences to plan and execute the ciclovía. Fourth, the steering committee reviewed the community stakeholders engaged in past ciclovías and reached out to other community members from their town and neighboring towns to promote awareness of ciclovía and its benefits.

### Data collection

The evaluation plan was guided by the toolkit developed by Hipp and Eyler (2015) (Open Streets Initiatives: Measuring Success Toolkit available on the Active Living Research weblink: (Open Streets Initiatives: Measuring Success Toolkit) [21].

#### Participant observation count form

The participant count form was adapted from similar forms used in nationwide bicycle/pedestrian counts, such as those done by Alta Planning + Design [22]. The form had specific categories to which the data collector assigned observed participants according to gender, child or adult, and activity (i.e., biking, walking, or other wheels) (see Additional file 2 for Count Form and Protocol). Such categorization helped capture how many people attended the event and how each participant was being physically active (e.g., biking in the open street, running in the soccer station). Counts were conducted by trained data collectors at multiple pre-identified stations. Each data collector was assigned a series of 15-min periods in which they counted the number of people that crossed their vision (as in crossing an imaginary line directly in front of them) and categorized them according to the criteria above. The data collector had one form for each time period. Each participant was marked as a tally mark, which were summed up per category and in total for each 15-min time period. Each 15-min count was multiplied by 4 at each hour, and the average attendance per hour was estimated by taking the mean across the duration of the ciclovía, in hours.

#### Participant intercept survey

The survey consisted of 16 questions in 2016, capturing information such as motivation to attend the ciclovía (open-ended question); past ciclovía attendance; the time the participant has spent, or plans to spend, on ciclovía activities during the ciclovía (walking, bicycling, activity station, running, other/specify); what the participant would have been doing if not at the ciclovía ((1) at home indoors such as watching TV, working on computer, reading; (2) other recreational activities indoors; (3) other recreational activities outdoors; (4) other/specify); and demographic information (see Additional file 3 for Survey). Additional questions were added in the 2019 survey to capture where participants heard about the ciclovía, their comprehension of the event, barriers to attendance, and perceptions of the benefits of ciclovía.

Data collectors were bilingual (Spanish and English) research staff and community volunteers previously trained on survey data collection. Data collectors were stationed in their designated locations and approached adults (> 18 years old) at the event, asking if they would be willing to participate in the survey. If the participant answered that they had completed the survey, then the data collectors moved to the next adult. Once the participant agreed to participate, the data collector administered the survey. The survey took 3–5 min to complete.

## Results

The progression of the research intervention into community adoption spanned 3 years (2016–2019). The planning committee was established in February 2017, and the first planning meeting took place in April 2017. The implementation of the first and second ciclovías took place in July 2017 and May 2018, respectively, and the community-adopted program took place in June 2019. All ciclovía dates were selected by the planning community.

### Participant observation count

**Fig. 1:**
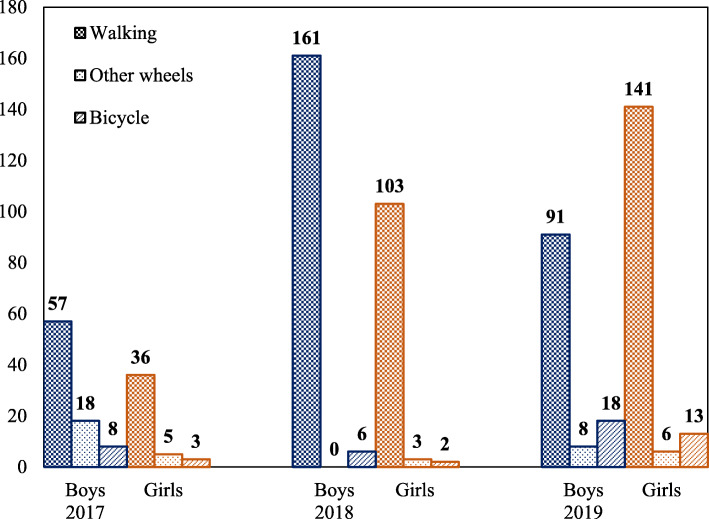
Attendance to ciclovía by gender among children across three years (2017–2019)

**Fig. 2:**
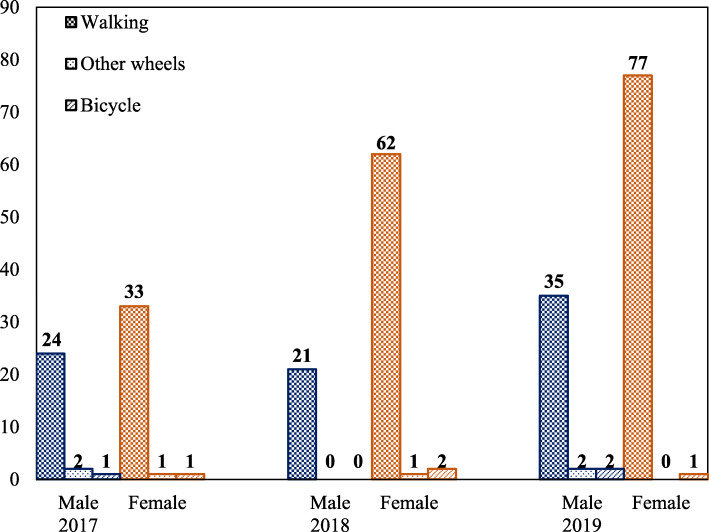
Attendance to ciclovía by gender among adults across three years (2017–2019)

Community attendance increased with each subsequent ciclovía. On average, 189 people (127 children and 62 adults) were present at each hour in 2017; attendance increased to 361 people/hour (275 children and 86 adults) in 2018, and to 394 people/hour (277 children and 117 adults) in 2019 (Figs. 1 and 2). Among children, more boys, compared to girls, attended ciclovía in 2017 (83 boys and 44 girls) and 2018 (167 boys and 108 girls), but more girls attended in 2019 (117 boys and 160 girls) (Fig. 1). Children on bicycles slightly decreased from 2017 to 2018 from 11 children (8 boys and 3 girls) to 8 children (6 boys and 2 girls) but increased in 2019 to 31 children (18 boys and 13 girls). Among adults, more women, compared to men, attended ciclovía throughout the 3 years: 27 men and 35 women in 2017, 21 men and 65 women in 2018, and 39 men and 78 women in 2019 (Fig. 2). Adults on bicycles remained low throughout the years; 2–3 from 2017 to 2019. Most adults were walking: 57 (24 men and 33 women) in 2017, 83 (21 men and 62 women) in 2018, and 112 (35 men and 77 women) in 2019.

### Participant survey

Participants’ reasons for attending ciclovía were wanting their children to experience ciclovía and seeing the promotion flyers. In 2019, they also reported wanting to learn more about health education. Participants reported that ciclovía should occur 2–3 times per year (Table 1). Many participants reported that they would have been staying home indoors if they did not attend ciclovía (65.60% in 2017, 76.20% in 2018, and 67.44% in 2019), and they planned to stay at the event for 1–2 h. Participants reporting attendance at previous ciclovías steadily increased: 12.5% in 2017, 19% in 2018, and 51% in 2019. Walking and visiting activity stations were consistently reported as the most preferred activities across the 3 years, with significant increases among those noting walking (34.38% in 2017, 42.71% in 2018, and 95.24% in 2019). A steady increase was also reported for the mean (+SD) number of days the participant reported being physically active (3.59 + 2.51 in 2017, 3.65 + 2.29 in 2018, and 4.33 + 2.27 in 2019).

**Table 1:** Resident and neighbordhood charactersitics of ciclovia participants from 2017 to 2019

	2017 (7/14/2017) ***N*** = 32	2018 (5/19/2018) ***N*** = 63	2019 (6/22/2019) ***N*** = 43
**Sex, n (%)**			
Male	8 (25.00)	9 (14.30)	5 (11.63)
Female	24 (75.00)	54 (85.70)	37 (86.05)
**Age, Mean (SD)**	38.88 (10.62)	39.83 (11.25)	43.33 (12.53)
**Household population, Mean (SD)**	4.81 (1.60)	4.87 (1.76)	5.14 (1.68)
**Household population (under the age of 18), Mean (SD)**	2.31 (1.66)	2.56 (1.58)	2.5 (1.41)
**Race, n (%)**			
Hispanic / Latino	29 (90.60)	54 (85.70)	32 (74.42)
Non-Hispanic White	1 (3.10)	4 (6.30)	2 (4.65)
American Indian or Alaska Native	2 (6.30)	5 (7.90)	9 (20.93)
Other	0 (0.00)	0 (0.00)	2 (4.65)
**Education status, n (%)**			
Less than high school diploma	12 (37.50)	28 (44.40)	15 (34.90)
High school diploma or GED	9 (28.10)	16 (25.40)	14 (32.60)
Some college or more (2018)	11 (34.40)	19 (30.16)	13 (30.23)
Missing			1 (2.30)
**Home city**			
Buena	0	4 (6.3)	1 (2.33)
Grandview	0	1 (1.6)	1 (2.33)
Granger	0	0	1 (2.33)
Mabton	0	1 (1.6)	1 (2.33)
Outlook	1 (3.10)	0	0
Prosser	0	0	1 (2.33)
Sunnyside	0	3 (4.8)	3 (6.98)
Toppenish	29 (90.60)	49 (77.8)	30 (69.77)
Wapato	0	1 (1.6)	0
White Swan	1 (3.10)	0	0
Yakima	0	1 (1.6)	0
Zillah	0	2 (3.2)	4 (9.30)
Missing	1 (3.10)	1 (1.6)	0
**Neighborhood facilities**			
Small Park	15 (46.90)	29 (46.00)	28 (65.10)
Large Park	20 (62.50)	34 (54.00)	15 (34.90)
Playground	12 (37.50)	34 (54.00)	25 (58.10)
Basketball Court	10 (31.30)	29 (46.00)	23 (53.50)
Swimming pool	17 (53.10)	18 (28.60)	14 (32.60)
Other^b^	2 (6.30)	8 (12.70)	2 (4.65)
**How often (times per year) should Ciclovía events occur?**			
0	3 (9.38)	0	4 (9.30)
1	2 (6.25)	3 (4.76)	6 (13.95)
2	12 (37.5)	30 (47.61)	17 (39.53)
3	4 (12.5)	12 (19.05)	4 (9.30)
4+	11 (34.38)	18 (28.57)	12 (27.00)
**Attendance of previous Ciclovía**			
Yes	4 (12.50)	12 (19.00)	22 (51.16)
No	28 (87.50)	51 (81.00)	20 (46.51)
**Other activities if you were not at Ciclovía**			
At home indoors	21 (65.60)	48 (76.20)	29 (67.44)
Outside of the house, outdoors	5 (15.60)	8 (12.70)	9 (20.93)
Other	6 (18.80)	14 (22.20)	5 (11.63)
**Planned time to stay at Ciclovía, Mean (SD)**	68.91 (41.03)	128.06 (56.13)	121.74 (47.23)
**Time spent (or will spend) at Ciclovía by activities, Mean (SD)**			
Walking	48.41 (34.48)	67.08 (42.71)	40 (95.24)
Bicycling	33.75 (30.92)	35.83 (27.28)	9 (20.93)
Activity Station	49.64 (45.85)	47.98 (29.70)	28 (65.12)
Other Wheeled Device	0	0	4 (9.30)
Running	0	21.67 (7.64)	5 (11.63)
Other	0	82.5 (66.52)	2 (4.65)
**Days of physical activities (past 7 days by yesterday), Mean (SD)**	3.59 (2.51)	3.65 (2.29)	4.33 (2.27)
**Average Time**^**a**^ ** spent per day for physical activities (past 7 days by yesterday), Mean (SD)**	77.93 (100.51)	75.24 (78.72)	140.58 (149.98)

^a^Three participants removed due to extreme values in their reporting of 8 h per day

^b^In 2017, other = 2 participants (1 track and 1 trampoline); in 2018, other = 8 participants (2 trail, 1 pod, and 5 none), in 2019, other = 2 participants (1 trampoline and 1 gym)

Mean age (SD) of participants attending ciclovía increased slightly throughout the years from 38.88 (10.62) years old in 2017, 39.83 (11.25) in 2018, and 43.33 (12.53) in 2019. Participants were mostly women (75% in 2017, 85.7% in 2018, and 86.7% in 2019) with 2–3 children ages 18 and younger and who self-identified as Hispanic/Latino. Participation among those who self-identified as American Indian noticeably increased from 2017 to 2019: 6.3 to 20.93%, respectively. Five neighborhood areas most frequently identified for PA were small park, large park, playground, basketball court, and swimming pool.

## Discussion

In this paper, we present the process of developing and progressing ciclovía from a research intervention to a community-adopted program for rural communities and the creation of tools that can be adapted and disseminated to other communities. Our study shows that engagement of the community is vital to the development of a community-level intervention as it enables customization to the setting, thus improving its success for adoption. The engagement of the community also facilitated understanding of community resources and proposed pragmatic and realistic steps in the ciclovía planning tools.

Community participation increased when ciclovía was led by the community as community-adopted programming. This is in part due to the increased outreach by the community to organizations outside of the intervention community as research contamination was not an issue. Interestingly, while the demographics of the non-Hispanic white participants remained similar throughout the years, the participation among American Indians noticeably increased, suggesting potential appeal to this community. The participation in the 2019 ciclovía was 394 people on average each hour, which is over 4% of the town’s population. About 30% of the surveyed participants reported that they were from neighboring communities. This rate of attendance is higher than ciclovías in urban settings where less than 1% of the population participated [8]; this suggests that ciclovías in rural areas have greater appeal and a higher draw for PA than in urban areas, despite the greater distances between rural communities. In rural areas, there are limited high-quality, accessible, and affordable places for physical activity, such as parks and recreation centers [23, 24], and ciclovías could provide opportunities for rural residents to engage in PA by taking advantage of the existing resources of the streets and opening them up for physical activity.

Our findings show that children are amenable to engage in more active transportation when opportunities are available. Similar to other communities, active transportation using bicycles was rare in this community. Bicycles donated by the community for children were raffled to families throughout the event to instill a connection between bicycles with the theme of ciclovía. In 2019, we saw approximately a three-fold increase of children on bicycles for both boys and girls demonstrating that community norms around active transportation can be created by increasing awareness and accessibility for active transportation. While adult use of active transportation remained low throughout the years, they helped establish a norm by enabling their children to bring their bikes to ciclovía. Interestingly, adults reporting time spent doing PA doubled in 2019. Three adults reported spending 8 h per day of physical activity. While this could be overreporting, removal of these three adults did not affect the differential increased observed in 2019.

Our ciclovía participants reported wanting 2–3 ciclovías per year. While ciclovías are open and free for anyone, organizing this event takes a considerable amount of effort by multiple community stakeholders, volunteers, donations, and strong leadership by many community members [7, 25]. Rather than creating multiple ciclovías per year, ciclovía planners may want to focus on disseminating the ciclovía to neighboring communities by sharing their knowledge and experience and by encouraging the use of the planning guide. Eyler and colleagues (2015) found that many groups or organizations that have been implementing open streets initiatives for multiple years have helped integrate open streets events into the city’s strategic plan, or facilitated the creation of an open streets strategic plan, thus sustaining its impact [25]. This strategy will ensure that multiple communities gain this knowledge and that ciclovías are regularly held and rotated across communities throughout the year; this will then spread the efforts and resources across communities while creating safe, accessible, and affordable places for PA in rural areas.

### Strengths and limitations

One of the strengths of this study was the community partnership early in the process, leading to the optimization of ciclovía to be used in a rural setting. Another strength of this study is the focus on understanding factors that lead to successful implementation, a question that has been previously posed in the literature [6]. There are several limitations to the study. First, overall attendance was estimated from three count stations. It is likely that some event attendees were counted multiple times; thus, attendance could have been overestimated. Estimating attendance is a challenge as there is no standardized and agreed upon method of making estimates, thus making comparisons with other cities’ events difficult [8]. Second, while we tried to control attendance to ciclovía, when it was a research intervention, to intervention children and their families by publicizing the event to the children in the intervention community only, survey data show attendance from families of neighboring communities, suggesting contamination in greater numbers in 2018. Third, response bias may have been introduced as survey respondents were self-selected to attend the ciclovía. Fourth, while our attempt was to survey as many adults as possible, they were coming and leaving ciclovía at different hours; therefore, survey respondents may not have been representative of all attendees.

## Conclusion

Children living in rural areas are less likely to be physically active compared to their urban counterparts, in part due to the lack of infrastructure and opportunities for physical activity. Open streets event initiatives where roads are temporarily closed to motorized vehicles can provide safe spaces for PA and become sustainable rural community infrastructure. Our study shows that a strong engagement from the community is vital to the development and the subsequent adoption of the ciclovía. Community interest, participation, and desire for more frequent ciclovías increased as community members experienced the benefit of ciclovía. As efforts to organize ciclovía require substantial human capacity and resources, ciclovía planners may want to disseminate the planning tools to neighboring communities and strategically rotate ciclovía across several communities to increase the benefit while spreading the resources. Future research needs to explore the dissemination of the planning tool as well as fidelity of the tool for communities that adopt the event.

## Supplementary Information

**Additional file 1.** Planning checklist.

**Additional file 2.** Count protocol and form.

**Additional file 3.** Participant Survey (English and Spanish).

## Data Availability

Data files and materials pertaining to this publication are available upon request at lindako@uw.edu.

## Abbreviations

STRIDE: Strategizing Together Rural Interventions for Diet and Exercise
SOP: Standard Operating Procedures
BMI: Body Mass Index
PA: Physical Activity
