# Supplementary material for: Participation and engagement of a rural community in Ciclovía: progressing from research intervention to community adoption
Source: BMC Public Health. 2021 Oct 30;21:1964. doi: 10.1186/s12889-021-11980-6 (PMC8556949; doi:10.1186/s12889-021-11980-6)
Supplement: Supplementary file 1 — Additional file 1. Planning checklist. [file 12889_2021_11980_MOESM1_ESM.docx]

| Activity | Equipment Needed  **Additional file 1 – planning Checklist** | Main Contact | Status | To Finalize (Responsibility) |
| --- | --- | --- | --- | --- |
| Publicity | - Flyers to schools - Flyers for stores - Community advertisement - Banner |  |  |  |
| Street Closure/Barricades | - Barricades - Signage |  |  |  |
| Portable Restrooms/Wash Stations | - Portable restrooms & handwashing station - Hand sanitizer - City trash bin |  |  |  |
| Prize Station:  Prize list – helmet for each set of wheels | - Prizes - Passports - Passport bin - Signage - Table/chairs |  |  |  |
| Information Booth/Volunteer station/First Aid Station | - Table/chairs - First Aid supplies - Signage |  |  |  |
| Volunteers | - Gear Up Kids - YAB members - SAD Program kids - Others? |  |  |  |
| DJ/Announcer (Public Speaker) | - Music equipment - Speaker system - Microphone |  |  |  |
| Water Station | - Table - Portable water jugs - Water dispensers/stands - Water pouches - Signage |  |  |  |
| Fruit Stand | - Table/chair - Trash can - Fruit - Signage |  |  |  |
| Face Painting | - Face paints - Cleaning supplies - Table/chairs - Trash can - Signage - Face painters (x2) |  |  |  |
| Health Organizations booths | - Tables/chairs - Sun canopy |  |  |  |
| Lawn Activities |  |  |  |  |
| Zumba | - Music - Specialized instructor - Signage |  |  |  |
| Aerobics | - Music - Specialized instructor - Signage |  |  |  |
| Walk the Plank | - Wood planks - Signage |  |  |  |
| Jump Rope | - Jump ropes - Signage |  |  |  |
| Hula Hoop | - Hula Hoops - Signage |  |  |  |
| Volleyball | - Volleyball net & ball - Signage |  |  |  |
| Soccer | - Table/chair |  |  |  |
| Obstacle Course | - Cones, rope |  |  |  |
| Corn Hole | - Corn hole target - Bean bags - Signage |  |  |  |
| Slip n Slide | - Slip n Slides - Water |  |  |  |
| Street Activities |  |  |  |  |
| Skateboard Tutorial | - Local skateboard volunteers |  |  |  |
| Bike Check/Maintenance | - Table/chair - Bike repair supplies |  |  |  |
| Bike Riding Course | - Chalk - Cones |  |  |  |
| Four Square/Hop Scotch | - Chalk - Bouncy ball |  |  |  |
| Fire Station water play | - Fire truck |  |  |  |
| Study Activities |  |  |  |  |
| Community Champion Nomination | - Nominee list to selected voting panel |  |  |  |
| Ciclovía Survey | - Hard copies of Survey - Hard copies of tally sheets & protocol |  |  |  |
