# Supplementary material for: Participation and engagement of a rural community in Ciclovía: progressing from research intervention to community adoption
Source: BMC Public Health. 2021 Oct 30;21:1964. doi: 10.1186/s12889-021-11980-6 (PMC8556949; doi:10.1186/s12889-021-11980-6)

**Additional file 2 – Ciclovía Count Protocol and Form**

Standard Operating Procedure

Observations will take place at **4 points** along the route. There will be **one observer** at each location.

**Location 1: By the railroad station and bleachers**

**Location 2: Just inside park by Toppenish Ave and Asotin**

**Location 3: End of gravel drive by the volunteer/first aid station**

**Location 4: End of gravel drive by the bike information station**

There will be **4 observation periods** of 15 minutes each, beginning at 1:30pm:

1) 1:30-1:45, 2) 2:00-2:15, 3) 2:30-2:45, 4) 3:00-3:15

The four observers at each observation point will count and record quantitative observations of different types of participants.

- Check that there are 3 pages (front and back; 1 for each 15-minute period you observe).
- Complete the top box (Location #, Location Description, Start-Time, End Time).
- Draw an imaginary line in your visual site
- Count all the children and adults that move across the imaginary line.
- Age and gender are based on the observers’ best approximation.
- Conduct the count for 15 minutes.
- Once count period is finished, enter totals for each period into “total boxes.”
- Return the forms to Leader at the end of each data collection period.

**One observer per location will record:**

- The # of adult (18 <) participants by gender, who are walking, bicycling, and wheels.
- The # of child (<18) participants by gender, who are walking, bicycling, and wheels.

Age (whether over/under 18) and gender are your best approximation.

Be sure to write exact location on the tally sheet.

**NOTE:**

- Pretend there is an imaginary line and count each bicyclist or pedestrian who crosses it.
  - It does not matter where on the street or sidewalk a bicyclist or pedestrian crosses the line.
- Count anyone on a wheelchair or electric scooter as “Other Wheels.”
- Count the number of people on bikes (if two people riding a tandem bike, count as 2 bicyclists).
- Special circumstances:
  - Two people on a bike – count as 2 bicyclists
  - Person walking while carrying an infant – count as 2 walking (1 adult and 1 child)
  - Person walking with two infants in a stroller – count as 3 pedestrians (1 adult and 2 children)
- Multiple pass situation: A multiple pass is when someone walks (or rides) through your intersection, then time goes by (say 5-10 minutes) and then they come through again.
  - In this case, you count them again.
  - If time goes by and they walk (or ride) by a third time, count them again.
  - If you see the same person more than 3 times, please notate that (but do not count).
  - Please be accurate
    - Remember that the goal is to be accurate, not to turn in high numbers.
    - Your numbers may be low at your location. That’s okay.

**Ciclovía Participant Count Form**


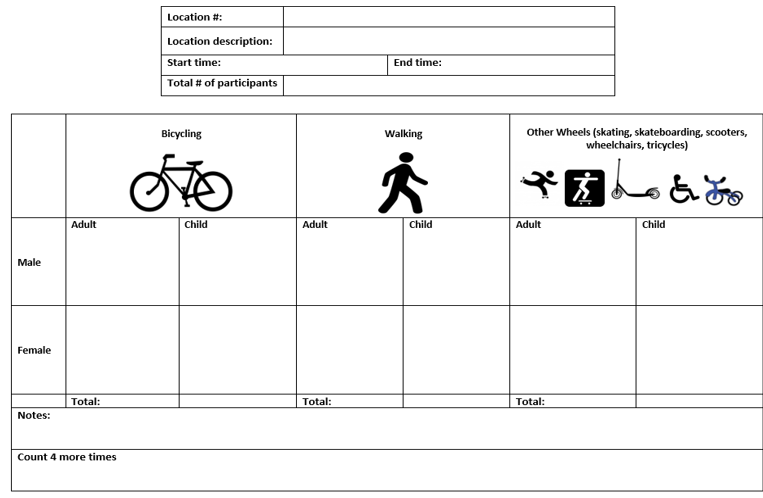

Supplement: Supplementary file 2 — Additional file 2. Count protocol and form. [file 12889_2021_11980_MOESM2_ESM.docx]
