# Supplementary material for: Participation and engagement of a rural community in Ciclovía: progressing from research intervention to community adoption
Source: BMC Public Health. 2021 Oct 30;21:1964. doi: 10.1186/s12889-021-11980-6 (PMC8556949; doi:10.1186/s12889-021-11980-6)
Supplement: Supplementary file 3 — Additional file 3. Participant Survey (English and Spanish). [file 12889_2021_11980_MOESM3_ESM.docx]

**Additional file 3 – Participant Survey**

**English Survey**

**By answering this survey, you are agreeing to participate in a research study that will help us understand how the community perceives today’s event called Ciclovía. Completing this survey is optional and NOT required to participate in this Ciclovía event. You can also skip any questions you are not comfortable answering.**

1. What is the reason you came to the Ciclovía event today? _____________________________________________________________________
2. Have you attended Ciclovía events in the past? □^1^ Yes □^0^  No
3. What do you think about this community event?
4. How often should Ciclovía events occur per year? ____ Times
5. What would you be doing if you were not here?

| □^1^ At home indoors (specify): ________________________________ |  |
| --- | --- |
| □^2^ Outside of the house, outdoors (specify):  _____________________________________________________ | □^3^ Other (specify):  ________________________________________________________ |

1. How long do you plan to stay here? ____ Hours ____Minutes
2. How much time have you spent or do you plan to spend doing the following activities today?

| Hours or Minutes | Hours or Minutes |
| --- | --- |
| □^1^ Walking: _____________ | □^4^ Other wheeled device: _____________________ _____________ |
| □^2^ Bicycling: _____________ | □^5^ Running: _____________ |
| □^3^ Activity Station: _____________ | □^6^ Other: ___________________________ _____________ |

1. Not including today, on how many of the past 7 days did you walk or do other moderate/vigorous physical activities (i.e., brisk walking, gardening, or anything that increases your breathing or heart rate)? ________ Days
2. During the past seven days, on average, how much time did you spend doing moderate/vigorous physical activity per day? ____ Hours ____Minutes
3. What is your home zip code? |__|__|__|__|__| What city do you live? _______________
4. Do you have any of the following in your neighborhood?

| □^1^ Small park | □^3^ Playground | □^5^ Swimming pool |
| --- | --- | --- |
| □^2^ Large Park | □^4^ Basketball Court | □^6^ Other: ___________________ |

1. What is your sex? □^0^  Male □^1^  Female □^2^  Other
2. What is your age? _______________
3. Including yourself, how many people live in your household? __________ Persons
4. How many are under the age of 18? _________ Persons
5. Which one or more of the following describes you?

| □^1^ Hispanic/Latino | □^4^ African American or Black | □^7^ Declined to answer |
| --- | --- | --- |
| □^2^ Non-Hispanic White | □^5^ Asian American and Pacific Islander |  |
| □^3^ American Indian or Alaska Native | □^6^  Other: ___________________ |  |

1. What is the highest level of education you have received?

| □^1^  Less than high school diploma | □^3^  Some college or more |
| --- | --- |
| □^2^  High school diploma or GED | □^4^ Other (specify): ___________________ |

**Spanish Survey**

**Al contestar estas preguntas usted está accediendo a participar en un estudio investigativo que nos ayudará a entender cómo la comunidad se siente acerca del evento de hoy llamado Ciclovía. Completar esta encuesta es opcional y NO es requerido para participar en esta Ciclovía. Puede saltar cualquier pregunta que no se sienta confortable contestando.**

1. ¿Cuál es la razón por la que vino hoy a la Ciclovía? _____________________________________________________________________________
2. ¿Ha ido usted a alguna Ciclovía antes? □^1^ Sí □^0^  No
3. ¿Qué piensa acerca de este evento comunitario?
4. ¿Cuántas veces deben ocurrir los eventos de Ciclovía al año? ____ Veces
5. ¿Qué estaría haciendo si no estuviera aquí?

| □^1^ En casa, adentro (especifique): ____________________________ |  |
| --- | --- |
| □^2^ afuera de la casa (especifique ):  ______________________________________________________ | □^3^ Otro (especifique):  ________________________________________________________ |

1. ¿Cuánto tiempo planea estar aquí? ____ Horas ____Minutos
2. ¿Cuánto tiempo ha pasado o piensa pasar haciendo las siguientes actividades hoy?

| Horas o Minutos | Horas o minutos |
| --- | --- |
| □^1^ Caminando: _____________ | □^4^ Otro aparato con ruedas: ____________________ _____________ |
| □^2^ Andando en bicicleta: _____________ | □^5^ Corriendo: _____________ |
| □^3^ En una estación de actividad: _____________ | □^6^ Otro: __________________________ _____________ |

1. Sin incluir hoy día, ¿en cuántos de los pasados 7 días caminó o hizo otras actividades físicas moderadas/vigorosas (ej. Caminar a paso rápido, hacer jardinería, o cualquier cosa que aumente su ritmo cardiaco)? ________ días
2. Durante los pasados 7 días, por lo general, ¿cuánto tiempo pasó haciendo actividades físicas moderadas/vigorosas por día? ____ Horas ____Minutos
3. ¿Cuál es el código postal de su casa? |__|__|__|__|__| En que ciudad vive usted? ____________________________
4. ¿Tiene algunos de los siguientes lugares en su comunidad?

| □^1^ Parque pequeño | □^3^ Patio de juegos | □^5^ Piscina |
| --- | --- | --- |
| □^2^ Parque grande | □^4^ Cancha de baloncesto | □^6^ Otro: ___________________ |

1. ¿Cuál es su género? □^0^  Hombre □^1^  Mujer □^2^  Otro
2. ¿Cuál es su edad? _______________
3. Incluyéndose a sí mismo(a), ¿cuántas personas viven en su hogar? __________ personas
4. ¿Cuántas de esas personas son menores de 18 años de edad? _________ personas
5. ¿Cuál de los siguientes mejor describe a usted?

| □^1^ Hispano/Latino | □^4^ Africano Americano o Negro | □^7^ Se negó a decir |
| --- | --- | --- |
| □^2^ Blanco, no hispano | □^5^ Asiático Americano o de las Islas del Pacífico |  |
| □^3^ Nativo Americano o Nativo de Alaska | □^6^  Otro: ___________________ |  |

1. ¿Cuál es el nivel más alto de educación que usted obtuvo?

| □^1^  Menos que un diploma de high school | □^3^  Algo de colegio o más |
| --- | --- |
| □^2^  Diploma de high school o GED | □^4^ Otro (especifique): ___________________ |
